# Predicting altered bone biomechanics in juvenile mice: insights from microgravity simulation, loading interventions, and Raman Spectroscopy

**DOI:** 10.1186/s42826-024-00207-5

**Published:** 2024-05-14

**Authors:** J. P. Berteau

**Affiliations:** 1 Department of Physical Therapy, City University of New York - College of Staten Island, New-York, USA; 2 New York Center for Biomedical Engineering, City University of New York – City College of New York, New-York, USA; 3 Nanoscience Initiative, Advanced Science Research Center, City University of New York, New-York, USA

**Keywords:** Juvenile mice, Simulated micro gravity, Raman spectroscopy, Bone, Fracture

## Abstract

**Background:**

Microgravity, a condition experienced in a spatial environment, poses unique challenges to the skeletal system, particularly in juvenile organisms. This study aimed to investigate alterations in bone biomechanics of juvenile mice due to unloading – that simulates microgravity in the laboratory—and the effects of a bone-loading intervention. We compared bone compositional and mechanical properties between 21-six-week-old C57Bl/6 from a control group (wild type) and a group that underwent a tail-suspension unloading protocol to mimic microgravity (MG). The second group (MG) experienced additional in vivo loading protocol (MG + LDG) on the right hind leg, where dynamic compressive loading was applied to the right knee using a custom-built loading device.

**Results:**

Our results show that after two weeks, we successfully induced bone alterations by (i) decreasing the energy dissipated before fracture and (ii) decreasing the yield and maximum stress. In addition, we showed that Mineral to matrix component [ν1PO4/Amide I], Carbonate to Amide [CO3/Amide I], and Crystallinity [1/FWHM(ν1PO4)] are strongly linked in physiological bone but not in microgravity even after loading intervention. While Crystallinity is very sensitive to bone deformation (strain) alterations coming from simulated microgravity, we show that Carbonate to Amide [CO3/Amide I] – a common marker of turnover rate/remodeling activity—is a specific predictor of bone deformation for bone after simulated microgravity. Our results also invalidate the current parameters of the loading intervention to prevent bone alterations entirely in juvenile mice.

**Conclusions:**

Our study successfully induced bone alterations in juvenile mice by using an unloading protocol to simulate microgravity, and we provided a new Raman Spectroscopy (RS) dataset of juvenile mice that contributes to the prediction of cortical bone mechanical properties, where the degree of interrelationship for RS data for physiological bone is improved compared to the most recent evidence.

## Background

Microgravity, a condition experienced in the spatial environment, poses unique challenges to the skeletal system [1, 2], particularly in juvenile organisms [3, 4]. For instance, recent evidence [5] depicted that rodents exposed to spaceflight experience notable reductions in whole bone mechanical indices, bone mineral density, and calcium content, leading to deficits in bone architecture, changes in bone mass, and alterations in tissue composition. These cumulative effects ultimately result in diminished bone strength among spaceflight animals. Nevertheless, information regarding juvenile mice and their tissue composition in this context remains limited.

To study microgravity, which induces disuse Osteoporosis [6], investigators used tail-suspended mice to make them experience the lack of mechanical stimulation and stress occurring in spaceflight or long-time bedrest. To treat it, mechanical action on the skeleton – using therapeutic exercises or mechanical devices – has restored the physiological bone homeostasis and decreased the fracture risk. For instance, compressive loading has been applied to the knee using a force-compression apparatus, which controls the force and frequency of the loading treatment [7]. In addition, skeletal loading has been shown to provide long-term benefits by preparing the skeleton to offset both the cortical and trabecular bone changes associated with osteoporosis from aging and menopause [8]. Recent evidence [9] from female adult rats—subjected to 21 days of hindlimb unloading—showed notable alterations in bone health such as: ( i) the femoral bone mineral density decreased in hindlimb unloading animals compared to the control group, (ii) deterioration in microarchitecture, particularly within the cortical compartment, (iii) changes in physicochemical properties, increased cortical porosity, and a reduction in deformation capacity and resistance to bone stresses. In addition, their results underscore the critical role of mechanical stimulation in maintaining skeletal integrity in female adults, highlighting that even a brief period of disuse can lead to microscopic changes in the bone matrix—evaluated using Raman Spectroscopy—elevating the risk of fractures. While most of these studies have investigated mature mice (8 weeks old) [10, 11], making it a valuable model for the development of loading protocol for adults [12, 13], there is currently no data that combines mechanical tests and compositional assessment on juvenile models. While recent investigation sheds light on the limitations and opportunities in preserving bone health during space travel, contributing to specific markers of bone composition integrity – like the ones obtained by Raman Spectroscopy – is necessary to track bone alterations. By unraveling the intricacies of microgravity-induced bone alterations in juvenile mice depicted by compositional markers, we aim to pave the way for informed strategies to maintain skeletal integrity in future space exploration endeavors.

This study aimed to investigate alterations in the bone composition and mechanics of juvenile mice due to unloading and the effects of a bone-loading intervention. We hypothesize that a mechanical loading intervention could prevent the installation of bone alterations—coming from unloading—in juvenile mice. We compared bone compositional and mechanical properties between immature mice at six weeks old from a control group (wild type) and a group that underwent a tail-suspension unloading protocol to simulate microgravity. The second group experienced additional in vivo loading treatment on the right hind leg, where dynamic compressive loading was applied to the right knee using a custom-built loading device. We obtained unique datasets from mice fibulae through (i) Compositional assessment with Raman spectroscopy and (ii) Macroscopic mechanical properties tests.

## Methods

### Experimental overview

**Fig. 1:**
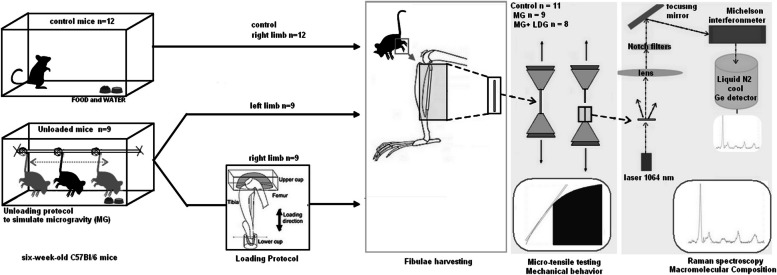
Experimental design overview

As depicted in the experimental design overview, we studied 21 six-week-old C57Bl/6 mice (Jackson Laboratory for Genomic Medicine, Bar Harbor, ME), as shown in the experimental design overview (Fig. 1). The 21 juvenile female mice were divided into two groups — *n* = 9 mice underwent an unloading protocol to induce juvenile OP, while we kept *n* = 12 mice in the Control group. We selected *n* = 9 for an acceptable coefficient of variability of RS parameters (Fiedler et al., 2021), where we added 30% for the risk of attrition (for bone harvesting and mechanical tests). We reduced to *n* = 9 for the Micro-gravity (MG) group after preliminary analysis of the control group and that the risk of attrition was limited. The experimental group underwent a loading treatment protocol on their right hind legs while we suspended their left hind legs, making the In vivo loading protocol (MG + LDG) Group. Finally, mice were sacrificed; 30 fibulae were harvested and divided into three groups: Control Group with fibulae (*n* = 12) that were harvested from the right limb of 12 control mice, MG Group with fibulae (*n* = 9) harvested from the left limb of the six mice that were unloaded with no loading treatment protocol, and MG + LDG Group with the fibulae (*n* = 9) harvested from the right limb of the six mice that were unloaded with the loading treatment protocol. The mice were (i) housed in our animal facility at a temperature of 20 °C to 24 °C, a 12/12-h light/dark cycle, and (ii) kept in individually ventilated cages with food and water ad libitum – for bedding, spruce wood shavings were provided. All materials were autoclaved before use. Investigators performing mechanical tests differed from those who performed the Raman spectroscopy, and the group number was kept known only to the Principal Investigator (J.Ph B). 20% body-weight reduction was an endpoint established for the study. Here, no adverse events were recorded. Here, the primary outcomes were the mechanical properties, and the secondary outcomes were the RS parameters. Because of the experimental design, it was difficult to control the cofounders. Protocols with research questions, key design features, and analysis plan were approved by the College of Staten Island's Institutional Animal Care and Use Committee.

### Unloading intervention

After mice were anesthetized using isoflurane (1–3% L/min O2), the proximal one-half of their tails were wrapped with orthopedic tape and reinforced by a cotton string [14]. The experimental group (*n* = 9) was suspended for two weeks (t = 14 days) at an approximate 30-degree downward tilt, suspending both their hind legs without sacrificing full use of their forelimbs and access to food and water [6]. we did this unloading protocol while the mice were anesthetized and monitored using isoflurane (IACUC protocol approved by the College of Staten Island IACUC board).

### Loading intervention

After the unloading treatment continued for two weeks (t = 14 days), a loading protocol was performed on the right hind limbs of the experimental group while the left hind limbs were suspended. After mice were anesthetized using isoflurane (1–3% L/min O2), the loading treatment used the Xpert4000, ADMET, and MTESTQuattro software, which applies a load of 9N to the knee with a frequency of 40 cycles at 10 Hz with a load-unload ramp of 0.15 s and pause duration of 10-s intervals [7]. The Xpert4000 apparatus underwent a customization process similar to that described in a prior publication concerning an osteoarthritis model, which we meticulously engineered for the precise administration of mechanical loads to bone samples (15). In this setup, a consistent force of 9 Newtons was applied to the knee joint of the mice, ensuring proper alignment under the activator. The loading process was synchronized with a frequency of 40 cycles per second, equivalent to a vibration rate of 10 Hertz. Throughout the loading–unloading sequence, a predetermined ramp pattern was followed, with both loading and unloading phases lasting 0.15 s each, designed to avoid cartilage disruption. Intervals of 10 s between cycles were incorporated to allow sufficient recovery and adaptation of the bone tissue to the mechanical stimuli. By meticulously implementing this controlled loading protocol, our objective was to mirror physiological loading patterns while minimizing undue stress on the bones, thereby facilitating a comprehensive exploration of the effects of mechanical loading on bone biomechanics under simulated microgravity conditions.

### Mechanical properties

Thirty fibulae were positioned vertically, aligned with the loading axis using a reference line behind the grips, and tested similarly. We previously tested fish bones [15, 16] using a micro-tensile tester (Xpert4000, ADMET, USA) customized with serrated pinching grips covered with carbide sandpaper to prevent samples from slipping (initial gauge length l_0_ = 3.0 mm and approximately 3.5 mm of both extremities being clamped). Samples were kept hydrated during the tests with drops of a phosphorus-buffered solution. The cross-section was measured using a Digital Caliper (Mitutoyo, 500–196-30, Japan). We applied uniaxial loading with a speed of 10 µm/s (quasi-static) while measuring the force with a load cell (100 N). Displacements were recorded regarding the change in actuator position Δl. We performed conversions from force (F) and displacement (l) to engineering stress (σ) and engineering strain (ε) according to Hooke's law with a custom-written Matlab script (MATLAB 2016b, Mathworks, MA, USA) [17, 18]. The following mechanical properties: yield strain and yield stress (${\upsigma }_{{\text{Y}}}$, ${\upvarepsilon }_{{\text{Y}}}$), max strain and max stress (${\upsigma }_{{\text{M}}}$, ${\upvarepsilon }_{{\text{M}}}$), plastic work (${{\text{W}}}_{{\text{P}}}$), elastic work (${{\text{W}}}_{{\text{E}}}$), total work (${{\text{W}}}_{{\text{T}}}$), and Modulus of Elasticity (MOE, Young’s Modulus) were derived from stress–strain curves as described previously in our published studies [15, 16, 19].

### Compositional properties

Raman spectroscopy was used to provide compositional information on the organic and Mineral components of the mice fibulae by using a 1064 nm FT-Raman spectrometer with a laser power of 150 mW (MultiRam, Bruker) and treating the data as previously described in our or colleagues publications [16, 20–22]. Fresh fibulae were analyzed at room temperature directly after the test, and the signal came from the surface of the fibula at the fracture site. Three consecutive acquisitions of 64 scans were performed. The laser irradiated the sample for 8 min for complete acquisition. We set the power of the laser at 150mW. The laser was polarized to control its direction of propagation. We chose the smallest resolution $$(1\mathrm{ c}{{\text{m}}}^{-1})$$ and collected the data from $$800\mathrm{ c}{{\text{m}}}^{-1}$$ to $$1800\mathrm{ c}{{\text{m}}}^{-1}$$. At the end of one acquisition, a spectrum is obtained. The spectra were analyzed using MultiRam software and our in house custom code. Compositional parameters were then investigated using a custom-written MATLAB script (MATLAB, MathWorks Inc., Natick, MA, USA) and a Peak Fitter version (Version 7.45, September 2015, Thomas C. O'Haver, University of Maryland College Park, USA). The mineral-to-matrix ratio [ν1PO4/Amide I] was obtained by dividing the phosphate peak (~ I959) by the Amide I peak (~ I1670). The carbonate-to-amide-I ratio [CO_3_/Amide I] was obtained by dividing the carbonate peak (~ I1072) by the Amide I peak. The carbonate-to-phosphate ratio was obtained by dividing the carbonate peak by the phosphate peak. Crystallinity was calculated using the reciprocal of the full-width-at-half-maximum of the phosphate peak [1/FWHM(ν1PO4)]. The Amide I region (from 1600 to 1720 $${{\text{cm}}}^{-1}$$) as a measure for the collagen phase was assessed by two methods: method (I) for Collagen crosslink/maturity ratio by dividing the subpeak at (~ I1660) by the subpeak at (~ I1690) and method (II) for its Collagen Integrity/Denaturation Status or Collagen Helical Status by dividing the subpeak at (~ I $$1670)$$ by the subpeak at (~ I $$1640)$$.

### Statistical analysis

Distribution was tested with the Shapiro–Wilk test; for testing differences between groups, one-way ANOVA was performed for normal distribution, while Kruskal–Wallis was used for non-normal distribution. The Pearson test was used to determine the correlation for normal distribution, while the Spearman test was used for non-normal distribution. Power sample studies were used to evaluate the risk of Type II error. We used SPSS Statistics (SPSS Inc., Chicago, Ill., USA).

## Results

**Fig. 2:**
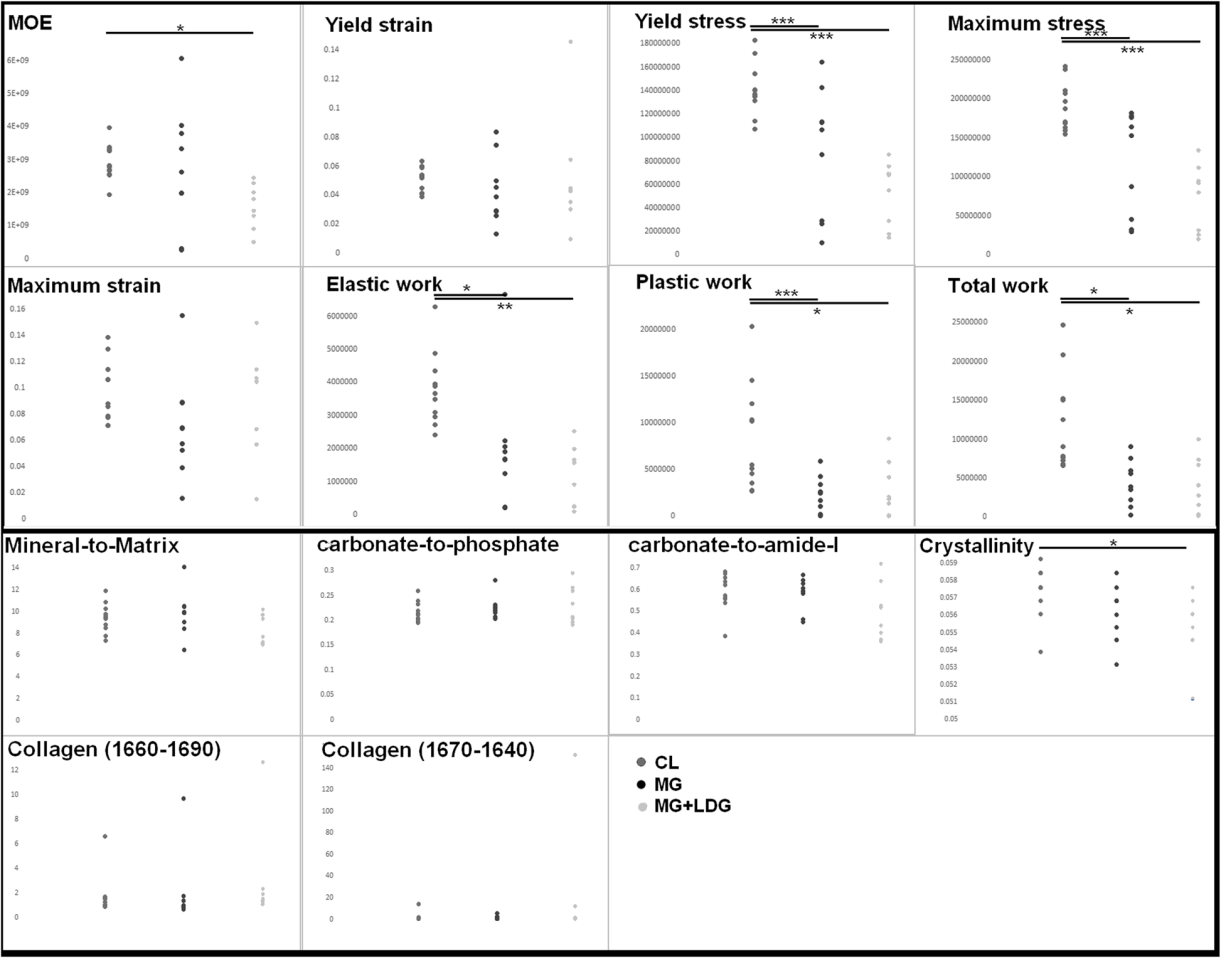
Biomechanical properties data (mechanical tests and compositional assessment through Raman spectroscopy) plotted for each group [Control, Micro-gravity (MG) and in vivo loading protocol (MG + LDG)] where significance is shown with * for *p* < 0.05, ** for *p* < 0.005 and *** for *p* < 0.001

The biomechanical properties' mean results ($\pm$ standard deviation) (mechanical tests and compositional assessment through Raman spectroscopy) are presented in Table 1, and all the data are plotted in Fig. 2. During the experiments, two fibulae broke when the mechanical tests started (one in the MG group and one in the MG + LDG group) because of misalignment. They were excluded from the data analysis.

**Table 1:** Mean results ($\pm$ standard deviation) of the biomechanical properties (mechanical tests and compositional assessment through Raman spectroscopy) for each group [Control, Micro-gravity (MG) and In vivo loading protocol (MG + LDG)] with data showing statistical significance in bold

	CL (*n* = 11)		MG (*n* = 9)		MG + LDG (*n* = 8)	
Mechanical Properties	mean	*SD (*+ */‐)*	mean	*SD (*+ */‐)*	mean	*SD (*+ */‐)*
MOE (Pa)	**2.90E + 09**	*5.20E* + *08*	2.71E + 09	*1.74E* + *09*	**1.59E + 09**	*6.32E* + *08*
Yield strain	5.11E‐02	*7.94E‐03*	4.33E‐02	*2.17E‐02*	5.22E‐02	*3.82E‐02*
Yield stress (Pa)	**1.41E + 08**	*2.11E* + *07*	**8.80E + 07**	*5.13E* + *07*	**5.20E + 07**	*2.57E* + *07*
Maximum stress (Pa)	**1.91E + 08**	*2.92E* + *07*	**1.17E + 08**	*6.30E* + *07*	**7.42E + 07**	*4.03E* + *07*
Maximum strain	9.70E‐02	*2.25E‐02*	7.06E‐02	*3.71E‐02*	9.02E‐02	*3.88E‐02*
Elastic work (Pa)	**3.79E + 06**	*1.05E* + *06*	**1.99E + 06**	*1.79E* + *06*	**1.16E + 06**	*8.54E* + *05*
Plastic work (Pa)	**8.37E + 06**	*5.41E* + *06*	**2.41E + 06**	*1.80E* + *06*	**2.99E + 06**	*2.71E* + *06*
Total work (Pa)	1.22E + 07	*5.90E* + *06*	4.40E + 06	*2.75E* + *06*	4.15E + 06	*3.34E* + *06*
Raman Parameters	mean	*SD (*+ */‐)*	mean	*SD (*+ */‐)*	mean	*SD (*+ */‐)*
mineral‐to‐matrix	9.42E + 00	*1.26E* + *00*	9.87E + 00	*1.92E* + *00*	8.51E + 00	*1.29E* + *00*
carbonate‐to‐phosphate	2.16E‐01	*1.90E‐02*	2.25E‐01	*2.12E‐02*	2.32E‐01	*3.57E‐02*
carbonate‐to‐amide‐I	5.86E‐01	*7.99E‐02*	5.81E‐01	*7.14E‐02*	4.97E‐01	*1.21E‐01*
Crystallinity	**5.74E‐02**	*1.39E‐03*	5.60E‐02	*1.60E‐03*	**5.53E‐02**	*1.82E‐03*
Collagen (1660/1690)	1.72E + 00	*1.58E* + *00*	1.98E + 00	*2.73E* + *00*	2.90E + 00	*3.70E* + *00*
Collagen (1670/1640)	2.25E + 00	*3.72E* + *00*	1.72E + 00	*1.49E* + *00*	2.13E + 01	*4.96E* + *01*

### Mechanical and compositional properties

Regarding differences between Control and MG, significant differences exist in the stress (ε_Y_, ε_M_) and all the energies dissipated (${{\text{W}}}_{{\text{E}}},{\mathrm{ W}}_{{\text{P}}}$, and ${{\text{W}}}_{{\text{T}}}$). The Indicative comparisons show a decrease of 64% and 37% for ε_Y_, ε_M_ respectively, and a decrease of 90%, 247%, and 176% for ${{\text{W}}}_{{\text{E}}},{\mathrm{ W}}_{{\text{P}}}$, and ${{\text{W}}}_{{\text{T}}}$, respectively. There were no statistical differences in the macromolecular composition. Between Control and MG + LDG, there were also significant differences for both stress and energies dissipated, with indicative comparisons showing a decrease of 170% and 157% for ε_Y_ and ε_M_, respectively, and a decrease of 226%, 179%, and 192% for or ${{\text{W}}}_{{\text{E}}},{\mathrm{ W}}_{{\text{P}}}$, and ${{\text{W}}}_{{\text{T}}}$, respectively. In addition, Crystallinity is significantly higher in the Control group than in the MG + LDG group, increasing by 5%. There was no statistical difference between MG and MG + LDG for mechanical and compositional properties.

Regarding the power sample study for the effect of the treatment intervention between MG and MG + LDG — for data that were normally distributed —, significance for MOE needs *n* = 29 MG and *n* = 12 LDG, significance for ${{\text{W}}}_{{\text{P}}}$ need *n* = 112 MG and *n* = 159 MG + LDG, significance for MTM between MG and MG + LDG needs *n* = 45 MG and *n* = 39 MG + LDG, for CTAI needs *n* = 43 MG and *n* = 37 MG + LDG, and for Crystallinity needs *n* = 160 MG and *n* = 158 MG + LDG.

### Statistical relationships

**Fig. 3:**
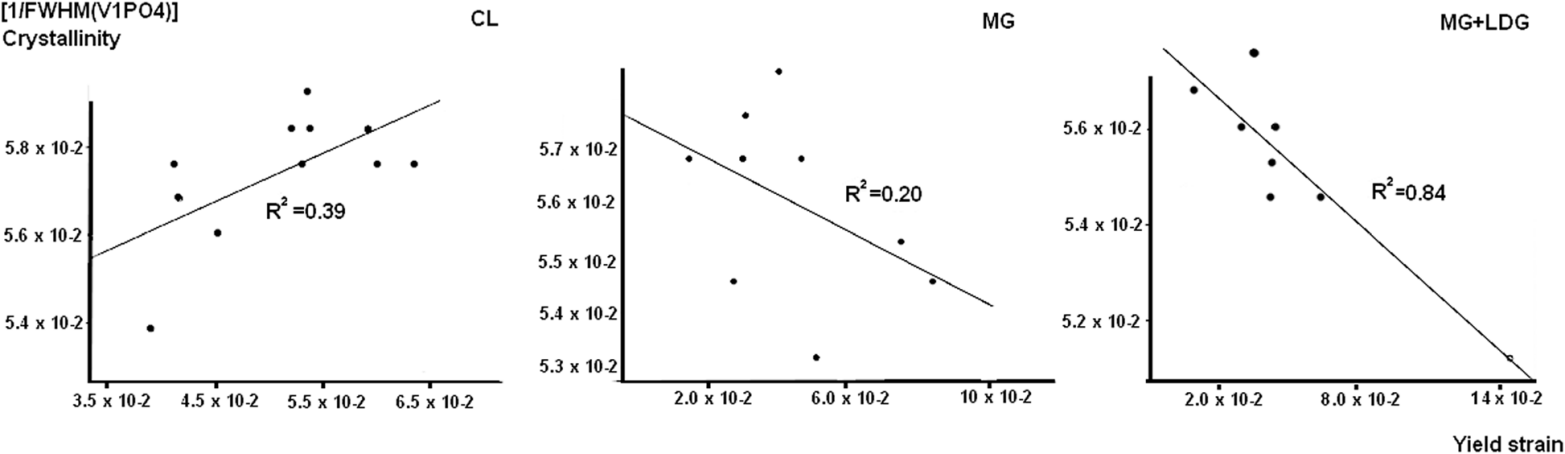
Parametric relationship (with R.^2^) between Crystallinity [1/FWHM(ν1PO4)] and yield strain ${(\upsigma }_{{\text{Y}}})$ for each group [Control, Micro-gravity (MG) and In vivo loading protocol (MG + LDG)]

**Fig. 4:**
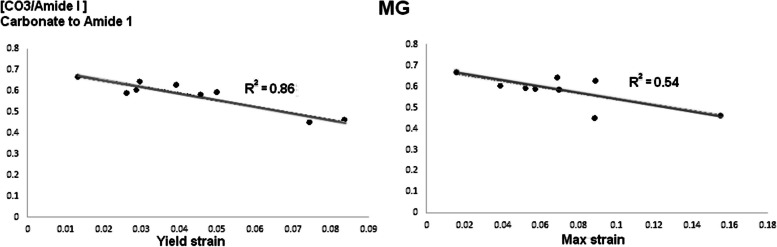
Parametric relationship (with R.^2^) between Carbonate to Amide [CO_3_/Amide I] and yield strain ${(\upsigma }_{{\text{Y}}})$ and max strain ${(\upsigma }_{{\text{M}}})$ for Micro-gravity (MG)

Regarding the relationship between Mechanical and Compositional Properties, we found a significant parametric positive relationship for the Control Group between Crystallinity and ${\upsigma }_{{\text{Y}}} {(R}^{2}=0.39$) (Fig. 3). We found a significant parametric positive relationship for the MG group between ν1PO4/Amide I & ${\upsigma }_{{\text{M}}}$ (${R}^{2}=0.657$). We found three significant parametric negative relationships between Crystallinity and ${\upsigma }_{{\text{Y}}} {(R}^{2}=0.20$) (Fig. 3), CO_3_/Amide I & ${\upsigma }_{{\text{Y}}}$ (${R}^{2}=0.86$), and CO_3_/Amide I & ${\upsigma }_{{\text{M}}}$ (${R}^{2}=0.54$) (Fig. 4). We also found a non-parametric positive significance between CO_3_/Amide I & ${{\text{W}}}_{{\text{E}}}$ (${R}^{2}=0.459$) and Collagen (1660/1690) & ${{\text{W}}}_{{\text{T}}}$ (${R}^{2}=0.115$). For the MG + LDG group, we found a significant parametric negative relationship between Crystallinity and ${\upsigma }_{{\text{Y}}}$ (${R}^{2}=0.84$) (Fig. 3). When we combined all the fibulae (control, MG, and MG + LDG), we found a significant parametric negative relationship between Crystallinity and ${\upsigma }_{{\text{Y}}}$ (Pearson Correlation ${R}^{2}=0.20$), as well as a significant parametric positive relationship between Crystallinity and ${\upsigma }_{{\text{M}}}$ (Pearson Correlation ${R}^{2}=0.18$).

**Fig. 5:**
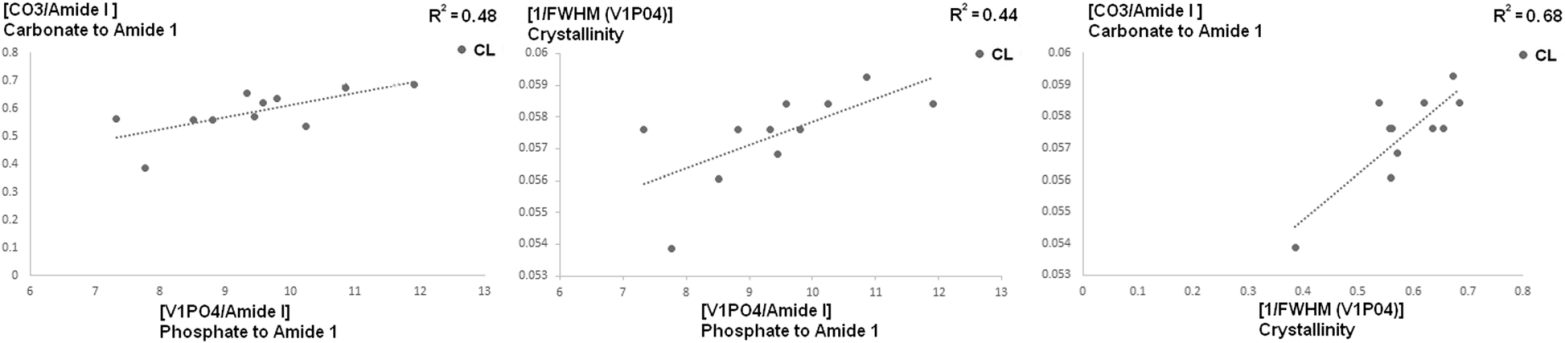
Compositional properties parametric relationship (with R^2^) between ν1PO4/Amide I, CO_3_/Amide I, and Crystallinity for the Control group

Regarding the Compositional Properties relationship, we have found for the Control group three significant parametric positive relationships between ν1PO4/Amide I and CO_3_/Amide I (${R}^{2}=0.48$), ν1PO4/Amide I, and Crystallinity (${R}^{2}=0.44$), and CO_3_/Amide I & Crystallinity (${R}^{2}=0.68$) (Fig. 5). However, we found no significance in the MG or MG + LDG group. Combining all the fibulae, we found two significant parametric positive relationships between Crystallinity and ν1PO4/Amide I (${R}^{2}=0.15$) and Crystallinity and CO_3_/Amide I (Pearson Correlation ${R}^{2}=0.33$).

## Discussion

This study furnishes compelling evidence that simulated microgravity induced by an unloading protocol, specifically tail suspension, reduces the energy dissipated before fracture in the fibula of juvenile mice. Moreover, our findings demonstrate that a bone loading protocol does not mitigate the extent of plastic deformation preceding fibula fracture during simulated microgravity. Consequently, our results show that juvenile mice subjected to simulated microgravity experience bone mechanical alterations beyond the preventive capabilities of our bone-loading interventions. It challenges the prevailing hypothesis that current mechanical loading protocols can effectively hinder the development of bone mechanical alterations in juvenile mice under simulated microgravity conditions.

Long-term evidence has depicted disuse osteoporosis – that can come from microgravity or bedrest—as caused by a simultaneous bone remodeling imbalance – an increase in bone tissue resorption (osteoclasts action) and a decrease in a new bone tissue formation (osteoblasts action) – and a decrease in the kinetics of remodeling processes. Thus, the cortical bone matrix has more time to mature and shows (i) an increase in mineral size [23], (ii) an increase in bone collagen crosslinking (both enzymatic and non-enzymatic) [17], and (iii) a decrease in water content [24] inducing both an increase in brittleness and a decrease in the plasticity of the whole bone. Recent evidence [5] depicted that in rodents subjected to spaceflight, there were significant decreases in whole bone mechanical indices, including a -15.24% difference in maximum load compared to ground control animals. Additionally, bone mineral density and calcium content were significantly reduced by -3.13% and -1.75% respectively. These findings suggest that spaceflight leads to significant deficits in bone architecture, a 6% loss in cortical area, and changes in bone mass and tissue composition, ultimately reducing bone strength in spaceflight animals. However, most of the time, tibia was the bone of interest. Previous evidence [25] has shown that the fibula displays similar adaptive responses to those previously documented in the tibia in mice (i.e., a synergistic increase in osteogenesis between loading and intermittent parathyroid hormone (iPTH) treatment), making fibula a suitable bone for the study of functional adaptation to mechanical loading. Thus, recent studies have investigated mice fibulae and how mechanical loading alters their biomechanical properties after hormonal changes during lactation [26] or fracture [27, 28]. On the contrary to previous evidence on the tibia [5], our unloading protocol shows changes in both ${{\text{W}}}_{{\text{P}}}$ and Crystallinity – a decrease in bone matrix plasticity with more mature crystals following previous literature [6, 12, 29]. However, our results indicate an increase in Crystallinity with lower ${{\text{W}}}_{{\text{P}}},$ which could indicate that parameters other than the mineral could have impacted the decrease of plasticity. In addition, our results show no significant differences between the MG group and MG + LDG group, neither in mechanical nor compositional properties. It suggests that the loading did not prevent MG. However, our results show a risk of type II error for MOE, ν1PO4/Amide I, and CO_3_/Amide I, where we could reach significance for groups of mice under 45 samples. It could indicate that a different type of bone loading – an increase in frequency, for instance – could reduce the risk of decreased mineralization, bone turnover, and elastic properties in a bone that underwent unloading.

Regarding the effect of the protocol we used here, previous studies on mice from 6 to 16 weeks of age [7] show that tibial stiffness and induced stresses from axial compression were consistently maintained, primarily due to growth-related changes in cortical cross-sectional geometry and longitudinal bone curvature. These factors had counteracting effects on bone stresses, highlighting the role of bone loading in preserving tibial stiffness during this developmental period. Although tissue mineral density increased slightly, its direct impact on tibial stiffness was minor within the examined age range. In addition, previous studies have focused on the trabecular bone to prove the validity of the loading treatment [30–32] by showing increased trabecular bone resorption in mice with osteoporosis after loading interventions (Sakata et al., 2009). However, most studies investigated the mice's tibia [30, 31], where mechanical tests are complex. Here, we investigated fibula mechanical properties – in a similar fashion, we used on other animal bones (intra-muscular and ribs fish bones) [15, 16] – using a tensile test where the highest strains occurred in the central portion of the specimen. In addition, evidence on mice fibula has shown that it is a sensitive bone to osteogenesis increases between loadings [25] making it an appropriate bone for research dealing with microgravity. A recent investigation [33] on age-related changes (mice 6, 12, and 22 months of age) has shown that alterations in mechanical strain response of mouse tibia and ulna, using axial compression tests, were different. Indeed, the tibiae showed no changes, while the ulnae showed a stiffer response with aging. Like the fibula, the ulna is a bone mainly consisting of a cortical component, while the tibia represents a mixed model of cortical and trabecular bone. In relation to this, our study supports this recent evidence on the ulna, and it suggests that the force or the frequency of the loading treatment protocol – which might be mainly effective on the trabecular bone – was insufficient to reduce bone alterations in our group of juvenile specimens. Despite the challenge posed by small bone sizes like fibula, the fact that limb bones experience the greatest forces in compression and bending modes highlights the potential benefits of investigating a three-point bending test in future research.

Assessing relationships between bone mechanical and compositional properties provides potential insights into bone alterations. Here, while we found—for the Control Group—a significant parametric positive relationship between Crystallinity and ${\upsigma }_{{\text{Y}}} {(R}^{2}=0.388$), the MG and the MG + LDG group show a negative relationship between Crystallinity and ${\upsigma }_{{\text{Y}}}$ (Fig. 3). In addition, for the MG group solely, Carbonate to Amide 1 ratio – a standard marker of turnover rate/remodeling activity [34]—seems to be a strong predictor of bone deformation (Fig. 4). Thus, in contrast to Carbonate to Amide 1 that seems specific to MG, our results suggest that crystallinity alterations – positively correlated in control to negatively correlated in MG and MG + LDG – could be a biomarker of a decreased strain deformation related to MG. However, recent evidence linking mechanical and macromolecular properties does not always agree. For instance, [35] showed that Crystallinity correlates with MOE, ε_Y_, and ε_M_ but not with strain parameters, and [36] showed that Crystallinity correlates only with yield strain. These differences could be explained by the different mechanical tests used. Indeed, while Unal (2021) used three-point bending tests and [36] used a Micro indentation test, we used a tensile test where the maximum tensile stresses are experienced throughout the entire volume of the bone sample. These discrepancies prevent us from clearly establishing the link between Crystallinity and bone mechanical properties. In addition, we found for the control group that 48% of the variance of ν1PO4/Amide I was explained by the variance of CO_3_/Amide I, and 44% of it was explained by the variance of Crystallinity (Fig. 5). Thus, our results improved the level of the relationship by providing a parametric interrelationship for RS data – where others [35] provided a non-parametric relation. In addition, we propose a novel relationship where Crystallinity explains 68% of CO_3_/Amide I variance. However, the level of relationship dropped when we combined all the fibulae – Crystallinity & ν1PO4/Amide I (${R}^{2}=0.148$) and Crystallinity & CO_3_/Amide I (${R}^{2}=0.332$) – suggesting that the unloading protocol altered the mineral properties of the cortical bone drastically. Although the study did not include micro-computed tomography analysis of cortical microstructure, the chemical composition and mechanical properties analysis provided valuable insights into the effects of unloading and mechanical interventions on bone structure. While future studies, including micro CT analysis, would allow for a more comprehensive evaluation of the relationship between bone cross-sectional geometry, 3D architecture, and bone fracture risk, the findings from this study contribute to our understanding of the bone structure and function in response to mechanical loading in MG.

While this study has provided valuable insights into the bone alterations induced by microgravity simulation in juvenile mice and the effectiveness of a bone-loading intervention, it is crucial to acknowledge certain limitations and consider future perspectives. For instance, future research endeavors could encompass extending study durations, amplifying sample sizes, and embracing a wider spectrum of age groups and species. Such steps would fortify the robustness and generalizability of our findings concerning the impacts of microgravity. Furthermore, integrating a meticulous examination of bone geometry and structure through micro-computed tomography (micro-CT) could furnish profound insights into the structural alterations induced by both microgravity and loading interventions. Indeed, the two-week duration of the study may present constraints in capturing the long-term effects of microgravity on bone health. Applying simulated microgravity in a tail-suspension protocol may partially replicate the complex gravitational conditions experienced in space. However, recent study findings [37] indicate that hindlimb suspension resulted in cortical and trabecular bone loss in mice, and concurrent exposure to low-dose-rate gamma irradiation did not worsen these bone loss effects. Thus, their results suggest that the mechanical unloading associated with suspension may substantially impact bone loss more than the low dose rate of radiation administered. Future investigations could explore refining and optimizing the loading parameters to develop more effective strategies for mitigating microgravity-induced bone changes. While we are missing geometrical and structural data in the bone studied, it paves the way for future investigations to incorporate a more comprehensive analysis of bone geometry and structure to directly assess the impact of loading on bone health in immature mice by using Micro-Computerized tomography. For instance, the identified Raman Spectroscopy parameters, particularly the interplay between Carbonate to Amide [CO_3_/Amide I] – a standard marker of turnover rate/remodeling activity—and bone deformation, open avenues for further exploration.

## Conclusions

Our study delve into the effects of an unloading protocol in non-mature mice, explicitly focusing on microgravity (MG) using Raman Spectroscopy. Despite successfully inducing MG in juvenile mice, our findings challenge the efficacy of an accompanying loading protocol in preventing mechanical bone alterations. The force or frequency of the loading treatment, possibly targeting trabecular bone, may not be sufficient to counteract bone biomechanical property changes in this context. Additionally, our study reveals a lack of solid interconnections among key Raman Spectroscopy parameters in MG, even after bone-loading intervention. While Crystallinity proves sensitive to MG-induced bone deformation, Carbonate to Amide [CO_3_/Amide I] emerges as a specific predictor of bone deformation in the presence of MG. In conclusion, this research identifies limitations in current intervention strategies for MG-induced alterations in juvenile mice. It contributes a novel RS dataset, enhancing our understanding of bone adaptation in microgravity environments.

## Data Availability

All datasets are presented in the main paper.

## Abbreviations

CL: Control
MG: MicroGravity
MG + LDG: MicroGravity with additional in vivo LoaDinG protocol
ν1PO4/Amide I: Mineral to matrix component
CO_3_/Amide I: Carbonate to Amide
1/FWHM(ν1PO4): Crystallinity
RS: Raman Spectroscopy
